# Current practices in the spatial analysis of cancer: flies in the ointment

**DOI:** 10.1186/1476-072X-3-22

**Published:** 2004-10-12

**Authors:** Geoffrey M Jacquez

**Affiliations:** 1 BioMedware, 516 North State Street, Ann Arbor, MI, 48104-1236, USA

## Abstract

While many lessons have been learned from the spatial analysis of cancer, there are several caveats that apply to many, if not all such analyses. As "flies in the ointment", these can substantially detract from a spatial analysis, and if not accounted for, can lead to weakened and erroneous conclusions. This paper discusses several assumptions and limitations of spatial analysis, identifies problems of scientific inference, and concludes with potential solutions and future directions.

## Introduction

'Dead flies cause the ointment of the apothecary to send forth a stinking savor; so doth a little folly him that is in reputation for wisdom and honour.'

Ecclesiastes 10:1

The term "flies in the ointment" is occasionally used to describe minor defects in some endeavor. But this quote from Ecclesiastes has a much wider scope than a few dead flies – it is the ointment itself that stinks, and the entire endeavor is thereby ruined. By analogy, there are several caveats that apply to many, if not all spatial analyses of cancer data. As "flies in the ointment", these caveats can substantially detract from a spatial analysis, and if not accounted or otherwise controlled for, can lead to weakened or erroneous conclusions. Several of these caveats have been identified in the papers in this collection; others have yet to be described. This paper brings them together in one location, where they are discussed under three broad headings.

• Problems of inference;

• Assumptions and limitations; and

• Potential solutions and future directions.

## Problems of inference: what can we learn from spatial analysis?

This section provides an overview of the scientific method as applied to spatial data, limitations inherent in the study of spatial systems, including those on inference, spatial methods and data, and finally, limitations imposed on spatial analyses of human health data by society and the context from which health data arise.

### Overview of the scientific method

*The Classic Paradigm of Karl Popper*. Popper [1] posed an approach to gaining knowledge from bodies of data that has come to be known as the "Scientific Method". Although his approach has been criticized as not necessarily being applicable to how scientific knowledge advances in practice, with fortuitous circumstance and flashes of insight (as occurred in the discovery of penicillin) receiving no mention, Popper's philosophy is useful because it incorporates inductive and deductive reasoning, and uses falsifiable predictions based on clearly stated hypotheses.

The useful lessons from Popper are first, that hypotheses and theories emerge from patterns and relationships in a set of observations; second, that the validity of the hypotheses is evaluated by distilling falsifiable predictions from them; third, that experiments designed to test these falsifiable predictions result in new data collected specifically to evaluate the predictions (these are designed experiments); and fourth, in order to avoid a tautology predictions cannot be tested on the data that gave rise to them. It is important to realize that useful predictions are falsifiable ones. Popper's approach can never prove a theory or hypothesis to be true. Rather, a body of evidence is collected from a series of experiments designed to test specific predictions, thereby increasing confidence that the hypothesis on which the predictions are based is true. Popper thus saw science as advancing much in the way used by Sir Arthur Conan Doyle's fictitious character Sherlock Holmes: If the alternative explanations are disproven, then the remaining explanation, no matter how unlikely, must be true.

Recognizing this, Platt [2], proposed what he called "Strong Inference". Strong Inference begins with a set of hypotheses regarding observed phenomenon. The researcher then designs a series of critical experiments to systematically test each hypothesis. Platt recognized that the set of alternative hypotheses may change during the course of the experimental process, and Strong Inference is thus more closely aligned with the experimental process as it is used in practice.

### Limitations inherent in the study of spatial systems

Spatial systems typically are large, and the spatial phenomena of interest in public health (e.g. cancer mortality rates, risk behaviors, demographic characteristics, and environmental exposures) are often difficult to observe directly and/or change slowly through time. This makes it difficult, if not impossible, to conduct designed experiments, and in any event there are substantial ethical considerations with experimentation on human populations. The spatial health researcher must often work with encountered data that have been collected for some purpose other than her specific study. In some instances the data are sampled in a systematic way from a spatially distributed population. But in each of these instances spatial analysis plays a critical role in identifying spatial and temporal relationships in population-level data, giving rise to hypotheses that can then be evaluated on additional data to be collected from the same system or on data from analogous spatial systems (spatial controls).

Under both Popper's and Platt's inference frameworks, study designs that attempt to confirm rather than reject hypotheses are not particularly useful. Repeating spatial studies to search for confirmation is less useful than undertaking analyses that are designed specifically to reject scientifically meaningful alternative hypotheses. But because it is so difficult to manipulate spatial systems, it can be difficult to design and undertake the critical analytical experiments that test falsifiable predictions.

### Limitations on inference

The spatial analyst's tool box includes techniques for quantifying spatial patterns, modeling risk surfaces, and assessing relationships between cancer outcomes and potential exposures. These techniques allow researchers to determine whether observed spatial patterns are statistically significant, to identify the locations of clusters, hotspots and cool spots, to construct maps showing excesses and deficits relative to a risk model, and to quantify association between two spatial variables (such as cancer incidence and putative environmental exposures). Although these techniques can be quantitatively powerful, the inferences that can be drawn from them have attendant limitations. We now consider three limitations on the inferences that can be reached from analyses (1) of spatial patterns, (2) of spatial associations, and (3) by using randomization (Monte Carlo)-based techniques.

*Pattern does not demonstrate causation*. As noted by Waller and Jacquez [3] tests for spatial pattern employ alternative hypotheses of two types; the omnibus "not the null hypothesis" or more specific alternatives. Tests with specific alternatives include focused tests [4] that are sensitive to monotonically decreasing risk as distance from a putative exposure source (the focus) increases. Acceptance of either of these types (the omnibus or a more specific alternative) only demonstrates that some spatial pattern exists, and does not implicate a cause. When the alternative hypothesis is highly specific, as for a focused test, it may correspond to a potential causal mechanism. For example, Waller et al [5] employed focused tests to explore a possible association between leukemia and lymphoma in New York State and exposure to TCE injected into ground water at industrial sites. While the score test employed was highly significant, demonstrating increased risk near several ground water injection wells, this finding did not demonstrate a causal relationship, or even that persons close to the injection wells had increased exposure to TCE. The existence of a spatial pattern alone cannot demonstrate nor prove a causal mechanism.

*Association is not causation*. The spatial analyst has an increasingly diverse suite of tools for documenting and quantifying associations between the spatial patterns of two or more variables. These techniques include cross-correlograms and related measures [6,7]., the bivariate LISA [8,9]., boundary overlap [10], polygon area overlap [11], as well as other approaches. Not intended to replace traditional statistical methods for association (such as the Pearson product-moment correlation), these methods assess the extent to which the spatial patterns in two variables (such as lung cancer incidence and ambient air toxic concentrations, see for example Jacquez and Greiling 2003) [12] coincide or "match up". But, as for traditional correlation techniques, a demonstration of spatial association does not demonstrate causality.

*Randomization limits inference to the data set*. Many disease cluster techniques and approaches to spatial modeling employ randomization, either based on sampling algorithms from spatial models (e.g. the Bernoulli model for the locations of cases and controls; the heterogeneous Poisson model for area-based cluster tests, and so on) or on distributional assumptions of randomization (e.g. the randomization hypothesis for Moran's I). Traditional statistics based on distribution theory (e.g. student's test, ANOVA etc) are able to make inferences regarding the "Universe" from which the population sample was drawn. Inferences for methods based on randomization, however, are limited *only *to the data set to which they were applied. This is one of the critical distinctions between methods based on distribution theory and "distribution free" techniques based on re-sampling a data set to construct empirical distributions [13].

### Limitations imposed by methods

All methods have attendant limitations, and this is true as well for techniques in the spatial analyst's toolbox. We now consider limitations imposed by spatial methods including the amount of knowledge required to use them, the selection and specification of spatial weights, and the subjectivity of the methods themselves.

*Amount of knowledge *Different analysis approaches require different amounts of knowledge. A distinction often is made between exploratory analysis, models of data, and models of process. When working with spatial data, a corresponding distinction can be made between Exploratory Spatial Data Analysis (ESDA), spatial data models, and spatial process models. Each of these (ESDA, models of data, and models of process) has different inferential/predictive abilities, and requires different amounts of data and knowledge of the spatial system itself. ESDA quantifies spatial pattern, models of data are used for interpolation and prediction, and models of process are used for prediction and the assessment of proposed perturbations to the spatial system. ESDA (including techniques such as autocorrelation analysis and disease clustering) aims to identify spatial patterns and to generate hypotheses that might explain those patterns. It requires relatively little knowledge of the system being studied. In fact, the objective of exploratory techniques is to explore and quantify relationships in order to increase the analyst's knowledge of the spatial system. Models of data (such as spatial regression, geostatistical models, risk surface models, and Bayesian techniques) require data of sufficient quality to estimate model parameters, and that the researcher possesses sufficient knowledge to be able to identify dependent and independent variables, and their relevant parameters. However the forms of these models do not convey any information regarding causal relationships. Models of process require a detailed understanding of the mechanics of the system being studied, and incorporate this understanding directly into the model itself. Spatial compartmental models that incorporate population and disease processes such as birth, death, migration and risk have been applied to model infectious diseases [14,15]. This kind of model has also been used to model the transport and fate of mutagenic compounds that are known carcinogens (e.g. [16]). But to date there are few if any process models that link population-level cancer outcomes to environmental exposures.

*Spatial weights *Each of the 3 types of approaches outlined above require the use and specification of spatial relationships among the objects (e.g. individuals, places of residence, areas of spatial support) being studied. In ESDA these are referred to as spatial weights. In models of data these may be called kriging weights (in geostatistics), autoregressive parameters (in spatial regression), or spatial filters (in Bayesian smoothing). In models of process spatial relationships are quantified to correspond to the underlying mechanics of the system, for example in an infection model, by how likely pairs of nearby susceptible and infectious individuals are to contact one another. As one moves from ESDA to models of process, the methods used for quantifying spatial relationships become increasingly meaningful in terms of the spatial system being studied.

*For ESDA spatial weights model the spatial disease pattern *(the alternative hypothesis). The selection and specification of spatial weights in ESDA is undertaken in the most "knowledge poor" circumstance, yet is critical since these weights quantify the alternative hypothesis of the pattern recognition statistic. For area-based data, commonly used spatial weights include first and higher order adjacencies, and functions of common border length. Some techniques evaluate nearest-neighbor and adjacency relationships on the centroids of areas, an approach that disposes of highly relevant geographic information (such as common borders) readily obtainable from polygon geometry. More advanced and realistic techniques are now being developed that account, not only for geographic relationships, but also for co-information such as population size [17]. But in general many of the spatial weights in common use are geographically crude (e.g. employ area centroids) and based entirely on Euclidean spatial relationships that ignore relevant co-information such as population size. Later in this paper we discuss the use of spatial weights to represent exposure mechanisms.

*Subjectivity *Most researchers recognize that all analytical methods impose a model, of one type or another, on the data and are therefore subjective. For example, the product-moment correlation coefficient imposes a linear model and is thus sensitive to linear relationships in bivariate data. Similarly, all techniques for spatial pattern analysis and modeling are founded on assumptions and are sensitive to or descriptive of different aspects of spatial pattern. For example, reliance on a single cluster statistic can only reveal those disease patterns that are consistent with that test's alternative hypothesis (e.g. circular or elliptical clusters for spatial scan statistics). This has prompted some researchers to employ a battery of spatial pattern methods to better describe different aspects of the morphology of geographic patterns in cancer incidence [12]. While employing a variety of techniques doesn't remove subjectivity, it does illuminate different aspects of spatial patterns, thereby providing a richer and more accurate description of geographic variation.

### Limitations imposed by data

The spatial data used in many geographic studies of cancer have inherent limitations attributable to granularity, spatial and temporal mismatch, under-reporting, misdiagnosis, the use of location as an exposure surrogate, human mobility, location and attribute uncertainty, static representation, as well as topological errors that result in erroneous spatial weights.

*Granularity *has to do with the spatial resolution of the data. For human health applications, death certificates are often georeferenced to location of place of residence at time of diagnosis or death. Point-based methods then use these coordinates directly. Area-based methods require the point locations to be aggregated to provide raw or adjusted rates within areas, and these areas might be census units, metropolitan statistical areas, counties, states and so forth. Because of the need to protect patient privacy, publicly available data are often aggregated to a sufficient extent to prevent the disclosure or reconstruction of patient identity. So, for example, point maps displaying patient place-of-residence typically cannot be disclosed by researchers and public health agencies. But due to the Modifiable Areal Unit Problem (MAUP) how these data are aggregated can dramatically impact analysis results, and incompatible geographies (e.g. census vs. ZIP Code) make tests for association problematic [18]. The ability to detect and model spatial pattern depends on granularity. One cannot, for example, detect clusters of counties using health data that is aggregated at the state-level. It is worth noting, however, that methods of spatial unmixing for raster-based data have been developed that support the construction of higher resolution maps from lower resolution information [19]. Unmixing approaches for disaggregating census and spatially aggregated health data that will allow spatial analyses using a common spatial support across variables are now available [20].

*Spatial and temporal mismatch *Cancer data, information on covariates and on environmental exposures typically do not "match up" in space or in time. For example, Jacquez and Greiling [12] analyzed lung cancer data on Long Island, and contrasted spatial patterns (geographic boundaries) with data on airborne toxics from EPA's (Environmental Protection Agency) National Air Toxics Assessment (NATA) program. Mismatch occurred between the cancer and air toxics data both in space (lung cancer incidence was reported at ZIP+4 level; air toxics data for census block groups) and in time (lung cancer incidence was reported for 1994–97; the air toxics data was based on emissions reported during 1996). The problem of spatial mismatch was solved by using spatial tests for association (boundary overlap) that account for the differing geographies within the randomization procedure. Temporal mismatch was problematic because latency for lung cancer is on the order of 15–20 years, and air toxics information could not be reconstructed over that time span. Thus while they found a positive geographic association between the air toxics and lung cancer incidence, the substantial temporal mismatch means a more detailed exposure reconstruction is required before any conclusions can be reached.

*Location and attribute uncertainty *Uncertainty in spatial health data occurs in two data components: the locations (e.g. coordinates of place of residence) and attributes (the values recorded at the locations). Also referred to as positional uncertainty, the impacts of location uncertainty on spatial pattern analysis and modeling have been well documented in the geographical and natural resource sciences [21,22]. In the health sciences, Jacquez and Waller [23] evaluated the impacts of location uncertainty on three tests for space-time interaction, and found the Mantel, Knox and k-nn tests to differ in their sensitivity to location uncertainty, with the k-nn test less likely to report false negatives as uncertainty increased. Location uncertainty can be modeled using several approaches, including lists of alternative locations for point-based data, and polygon, population, and risk-based models for area-based data [24]. Nonetheless, many spatial analyses of cancer assume locations are known with 100% certainty and that the spatial weights calculated from those locations are precise and without error in either representation (e.g. is it reasonable to use place of residence to represent human activity patterns?) or measurement.

*Location as an exposure surrogate *Location uncertainty has different sources, one of which is human mobility. Attempts at describing such mobility that transcend the use of place-of-residence to represent location include daily activity spaces[25,26], and constructs such as time geography and pathogenic paths [27-29]. But while almost all researchers acknowledge that many causative exposures occur outside of the home, most spatial analyses still rely on place-of-residence to georeference locations of health events. When might place of residence reasonably be used to georeference health data? For infectious diseases exposure events require contact between infected and susceptible individuals of sufficient duration to allow the pathogen to pass from one to the other. The exposure route varies from one type of pathogen to another, and a given pathogen may have several exposure mechanisms. These includes fecal-oral (e.g. the Norberg virus that recently has been the bane of cruise ships), intimate sexual contact (e.g. STD's and HIV), air-borne droplets (e.g. tuberculosis), and contaminated foods (e.g. hepatitis), among other mechanisms. Zoonotic and vector-borne diseases involve an animal host or reservoir, and exposure mechanisms may include animal-human as well as human to human routes. Spatial weights for such exposure routes may incorporate measures of geographic proximity, but also should be constructed to reflect the probability of exposure between pairs of individuals (for individual-based models) and for groups (for population-based models). Although exposure routes for infectious diseases are numerous and often quite complex, exposure reconstruction for cancers with long latency and for which mechanisms of carcinogenesis are only partially known is even more problematic.

*Use of place-of-residence in spatial analyses of cancer, and calculating purely spatial weights from those locations, seems appropriate only when individuals have resided at that location for as long or longer than the latency period, and when potential causative exposures occur either in the household or in the surrounding neighborhood*. For what cancers might causative exposures occur in the home? Lung cancers attributable to household radon are a good example, as well as cancers caused by combustion by-products from cooking and second-hand smoke. Cancers of childhood reasonably may use place-of-residence as an exposure surrogate since the latency period is short and children tend to stay near the home. For other cancers and at larger scales of aggregation, such as census and ZIP Code geography, human mobility, especially in commuter communities, poses a substantial challenge to spatial analysis of cancer, and the finding of a geographic cluster can thus be difficult to interpret when place of residence is used to represent locations of individuals. Recently, Meliker et al [30] used the constructs of time geography within a space-time information system to undertake the space-time modeling of individual-level exposure to arsenic. They were able to reconstruct individual arsenic exposure based on specific assumptions regarding occupational exposures and the ingestion of arsenic in drinking water. The time-geographic approach appears to provide a robust quantitative foundation for exposure reconstruction that is not possible when a single location is used to represent an individual's location in space-time.

*Under reporting and misdiagnosis*: Uncertainty in the attributes (e.g. case identifiers and the numerators in incidence and mortality rates) arises from under reporting and misdiagnosis. Under reporting is especially an issue when working with data that encompasses health districts with different recording and reporting practices. Because states maintain their own cancer registries, differences in reporting practices can pose a special problem for data sets that cross state boundaries. For most cancers, diagnostic accuracy decreases as one works with retrospective data when the physician's diagnostic arsenal was not as robust. In addition, classifications of disease change through time, as when the International Classification of Disease (ICD) code is updated. When either differences in reporting and diagnosis are present, once cannot preclude the possibility that observed spatial variation in cancer rates is attributable to these causes.

*Static view*: GIS typically represent the world as "snapshots" in time and do not effectively represent temporal change [31]. The importance of time in health geography is well recognized, since almost all geographic disease patterns are the result of space-time processes [32]. There thus are substantial limitations that arise from using conventional GIS technology, especially for the mapping, representation, and analysis of health, socioeconomic, and environmental information for populations that are dispersed or mobile and in which space-time relationships are dynamic. Advances in space-time information system technology address this deficiency using space-time coordinates and object representations that include motion and morphing, as well as attribute change models [30].

*Polygons, Topology and computational geometry*: The spatial analysis of area-based data requires the calculation of statistics such as polygon contiguity, length of common boundaries, areas and centroids. Calculation of these statistics employs methods of computational geometry that assume the polygons are correctly represented in the Euclidean plane. These assumptions usually are that polygons are closed (e.g. Jordan curves), and are not folded or joined together at single points to form "bow ties". When these assumptions are not met, techniques such as polygon triangulation will either fail or yield incorrect results, and resulting statistics, including placement of area centroids and spatial weights, will be wrong. Despite the importance of this problem, most spatial analysis software does not check shapefiles (which lack topological information) to determine whether the polygons are topologically well-conditioned. While this may seem an arcane problem, we have discovered in practice that a substantial proportion of the shapefiles shared among researchers for use in spatial analysis are flawed to a sufficient extent so that the resulting spatial weights are incorrect. One example is the primary care service area file  that has 60 of 6000 polygons self intersecting, a 1% error rate.

### Limitations imposed by society and context

*Limitations on inference in cluster investigations*. Many disease cluster investigations are initiated by reports from concerned citizens, and the attendant increase in the probability of false positives due to such preselection bias is well known [33,44]. Others have pointed out that the investigation of preselected clusters is not a scientifically valid endeavor [34], because of the tautology of testing hypotheses on the data from which they emerged, as well as other reasons. Several authors [35] have noted limitations of the hypothesis-testing framework relative to a more flexible spatial modeling approach. Nonetheless, it is the mission of public health departments to respond to public health concerns [36], and cluster investigations are likely to continue to be undertaken within a hypothesis testing framework such as that advocated by the Centers for Disease Control [37].

*Limitations arising from lack of communication with community stakeholders *Within public health departments spatial analyses of cancer data are best undertaken by teams comprised of a community stakeholder (e.g. community end-user of the study results), a political decision maker whose constituency is the subject of analysis, a public health practitioner capable of putting in place an intervention should the results be positive, a spatial analyst with a detailed understanding of the spatial analytic methods, and a GIS specialist to manage data and undertake mapping tasks. Such a team effort is most likely to translate analytical results into community action [43].

*Information democracy vs. protection of privacy Efforts *such as the National Spatial Data Infrastructure project are leading to the advent of data portals designed specifically to facilitate sharing and dissemination of spatial information. The DataWeb  is a network of online data libraries created in a collaboration between the CDC and the US Census Bureau. The libraries consist of both microdata and aggregate data, and include census, economic, health, income and unemployment, population, labor, cancer, crime, transportation, family dynamics, vital statistics, and other georeferenced data. Information in DataWeb is accessed through DataFerret, an application that prepares data sets for the user to download. It allows users to select a databasket of variables and then recode those variables as needed. Users develop and customize data tables and download them to their desktop (download formats include ASCII, SAS, SPSS, and Excel/Access).

Launched on June 30th, 2003, the Geospatial One Stop program  is a web-based portal for one-stop access to maps, data and other geospatial services that simplifies access to geospatial data collected by government agencies and other organizations. Geodata.gov is accelerating development and implementation of the National Spatial Data Infrastructure (NSDI) and includes state, local and tribal governments along with the private sector and academia as data providers. Geodata.gov offers access to thousands of data bases in 17 categories.

The promise of these and like efforts is an information democracy in which all citizens have ready access to information describing health, the environment, services, resources, the economy and other data, both for their immediate neighborhood as well as larger areas. While freedom of information is arguably one of the pillars of a democratic society, the need to protect individual privacy is a substantial countervailing consideration. There are other important ethical considerations with the sharing of spatial data of very high resolution. For example, satellite imagery is now publicly available at 1 m and sub 1 meter spatial resolution, and hyperspectral imagery at comparable resolution will soon be available. From such imagery it will be possible to identify and classify microhabitat for disease vectors [38], and even to map the transport and fate of heavy metals in rivers and streams [39]. But such information also will allow even small pockets of microhabitat of economically valuable species to be targeted for exploitation (for example, in the U.S. there is a black market in native endangered frogs, turtles, snakes and lizards). How will the need for spatial data sharing consistent with an information democracy be balanced with individual rights to privacy and related ethical considerations?

## Assumptions and limitations of spatial analysis of cancer data

Every study is based on assumptions, and ideally these are made explicit when the results are reported. This section describes several assumptions and considerations typical of spatial analyses of cancer, including ability to infer causality, the ecologic fallacy, and the role of higher-order interactions. This section concludes with a discussion of the lack of utility of the word "cluster" as a spatial pattern descriptor.

### Power to disprove but not confirm causality

Earlier we pointed out than an important limitation of spatial analyses of cancer data is that demonstration of significant geographic patterns and associations is *never *sufficient to demonstrate causality. This is particularly true of cancers because of long latencies, the substantial difficulties posed by exposure reconstruction, and because of our lack of a full our understanding of the environmental bases of carcinogenesis. However, the exploration and modeling of spatial cancer patterns can disprove predictions based on causal hypotheses that are expressed in spatial terms. For example, hypothesized exposure mechanisms that involve proximity to point sources, or for which attributed risks vary geographically, can be evaluated systematically using spatial analytic approaches.

### Ecologic fallacy, and arbitrary spatial partitions

Studies of geographic clusters and cancer data must include consideration of the potentially misleading aspects of ecologic studies. Even ZIP Codes and census tracts can be considered coarse spatial units for aggregating cancer cases and for estimating exposures, and exposure and health data often are not available at the same resolution. Exposure data often are reported at spatial levels, such as census tracts, that partition the geography in a manner inappropriate for the exposure process. Other spatial divisions may be better descriptors of environmental exposure, including, for example, watersheds, aquifers, local public water systems for water-borne substances, or "windsheds" for airborne substances. But because of privacy concerns for the patients and the limitations on existing environmental data, the data used often are simply the data that are available. Every data collection protocol has a design. Is it appropriate to use data for purposes other than for which they were collected? ZIP Code, census tract or place of residence at diagnosis is an inadequate descriptor of an individual's location during the development of cancer. For example, using the ZIP Code of residence assumes the patient lived within that ZIP Code area during the period of time required to develop cancer following exposure to an environmental compound that influenced cancer risk. Hence the degree of exposure to the potential risk factors over a multi-year period has been estimated for each study subject based on their place of residence, aggregated at the census tract level. This assumption is clearly tenuous given the mobility of human populations and the arbitrariness of the spatial partition for the environmental data [45].

In the 1980's many epidemiologists considered ecologic studies likely to lead to erroneous conclusions, and that the most accurate findings arise from individual-level data. Since the late 1990's, however, the potential of adding "contextual variables" to multi-level analyses has provided a sound methodological mechanism for combining individual-level data with higher geographical contextual data. Nonetheless, issues regarding the definition of spatial partitions, patient privacy, and the appropriate use of data still pertain.

### Higher order interactions

Especially for complex relationships (such as those between environment, genetics and cancer), apparent bivariate associations may be driven by multivariate interactions that are not directly quantified by the two variables under scrutiny. For example, elevated air pollution may be associated with lower housing prices (because of proximity to industrial sites), which in turn attracts poorer households with higher smoking rates. In this instance, an observed bivariate correlation between air pollution and cancer would actually overestimate the degree of association between these two variables. But because of their complexity, higher order multivariate interactions are difficult to quantify in spatial cancer studies.

### The term "cluster" has little meaning

The term "cluster" by and of itself is so generic as to be almost meaningless for describing spatial variation in cancers. What is a cluster? Is it an excess of cancer, and, if so, how much extra is considered an excess? Do we use likelihood statistics to find an excess, or should we use some other statistical framework? Are we looking globally to identify clusters anywhere in the study area, or do we define patterns locally, or relative to a putative source? These kinds of questions suggest that the declaration of a "cluster" is meaningless without a precise description of the statistical test being employed and the patterns to which the test it is sensitive. Because different clustering techniques are sensitive to different aspects of cluster morphology, analytic approaches that employ several pattern recognition methods can be more informative, especially in the ESDA phase of an analysis, with the caveat that the multiple tests will need to be accounted for should accurate estimates of P-values be required. Analyses that rely on just one kind of cluster test are incomplete in the sense that they will have power to detect only one type of cluster. Cancer morbidity and mortality evinces rich geographic variation, and it thus can make sense to employ a variety of techniques to more fully describe relevant aspects of spatial pattern.

## The future

This last section discusses salient trends and methodological challenges in the changing landscape of the spatial analysis of cancer. It summarizes expected improvements in cancer data, exposure measures, and genetic information, and concludes with some anticipated methodological and technological challenges for the next decade.

### Improved availability of cancer data

Recent trends in cancer registries are resulting in improved reporting and linking of spatially referenced data, although there is substantial variation in quality from state to state. There is a trend towards increased availability of aggregated cancer statistics over the World Wide Web. Not withstanding the inherent limitations of ZIP Code data, a good exemplar of improved availability is New York State, which is publishing online atlases of cancer incidence at ZIP Code level geography. A second example is the National Cancer Mortality Atlas published by the National Cancer Institute. New York State also makes available findings from spatial analyses of the cancer incidence data using the spatial scan statistic [40], and the National Cancer Atlas provides a narrative interpretation of the cancer mortality patterns and their potential causes. In coming months and years the quality of and speed with which cancer incidence and mortality data are made available is expected to improve, with some of these benefits attributable to improved Public Health Surveillance infrastructure currently being funded by bioterrorism and first responder initiatives.

### Improved exposure and population data

Efforts cited earlier in this paper such as the CDC's dataweb and geospatial onestop are making georeferenced information on socioeconomic, census, environmental, remote sensing and other data readily available for downloading over the web. In remote sensing, the trend is towards higher spatial, spectral and temporal resolutions which together hold great promise for improving environmental risk assessment, habitat classification, and change detection [41]. Modeling efforts by organizations such as the Environmental Protection Agency are integrating exposure models with spatial models of air-borne and other toxins, incorporating both point and non-point source information. Coupled with improved data on cancer health outcomes, enhanced exposure estimates, along with detailed descriptions of the affected populations, hold the promise of more detailed, accurate and predictive spatial modeling of cancer outcomes. However, this promise can only be realized when the data obtained from disparate sources is temporally matched, aggregated in an appropriate fashion, and collected at compatible geographic granularities.

### Improved genetic data

The recent revolution in gene sequencing, bioinformatics and proteomics is making possible a detailed understanding of genetic predispositions as well as the cascade of genetic changes that cause normal cells to turn into cancer cells. Research currently underway at the NCI is seeking to elucidate gene-environment interactions and how these interactions can lead to cancer, but in general spatial analysis has contributed little to the study of gene-environment interactions. In fact, such studies would require population-level information on genetic profiles and biomarkers sufficient to calculate human genetic distances, and this kind of data are not yet available. Some research has conducted on European populations to explore relationships between genetic distances calculated from blood polymorphisms and differences in cancer mortality [42]. But to fully exploit the potential of spatial analysis for the study of gene-environment interactions, more detailed data on the genetic profiles of human populations in the United States is needed.

### Improved technology

As noted earlier, the static view of GIS makes it difficult to represent human mobility and temporal change in cancer, environmental and socioeconomic data. GIS typically are based on spatial data models that apply to static spatial systems such as those found in geology, forestry, and physical geography. However, this purely spatial data model inadequately characterizes the "what, where, when" needed to effectively analyze cancer data and health-environment relationships. GIS built on spatial, rather than space-time data structures, cannot deal readily with space-time georeferencing nor space-time queries [31], and instead are best suited for analyzing static systems. Loytonen [32] and others have called for a "higher-dimensional GIS" (a Space-Time Information System or STIS) to better represent space-time dynamics. STIS provide a rich framework for the generation and evaluation of epidemiologic hypotheses founded on the exploration of space-time disease patterns in relation to their putative causes and covariates [9]. The advent of mobile computing and location-based services provide substantial opportunities for increasing our understanding of human activity patterns, and an important challenge for the spatial analysis of cancer will be to more fully exploit the temporal dimension as this information becomes more readily available.

### The methodological challenge

In the near future we will need techniques and methods that take full advantage of the burgeoning data stream while maintaining the values and ethos of an open, democratic society. Information detailing place of death, genetic makeup, socioeconomic status, product use, and lifestyle indicators will be available at unprecedented spatial and temporal resolution. Using these data, substantial benefits to society are expected to accrue from the rapid identification of cancers and other health risks. Syndromic and health surveillance systems are now being deployed that could make it possible to rapidly identify local increases in cancer risk, and even relate them to spatial patterns and changes in environmental data thought linked to causative exposures. But the benefits of analyzing such high spatial and temporal resolution data must be balanced against the need to maintain individual privacy, while at the same time providing equitable information access to all strata of society. Certain aspects of this problem can be met by the development of appropriate analysis techniques. Coming up with these techniques and applying them in a responsible fashion is a substantial challenge that will require the cooperation of researchers, funding agency program managers, and legislators.

## Note

The author is President of a commercial company (BioMedware) that develops software for the exploratory spatial and temporal analysis of health and environmental data.
